# Epigenetic regulation of H3K27me3 in laying hens with fatty liver hemorrhagic syndrome induced by high-energy and low-protein diets

**DOI:** 10.1186/s12864-024-10270-w

**Published:** 2024-04-16

**Authors:** Yong Cui, Meng Ru, Yujie Wang, Linjian Weng, Ramlat Ali Haji, Haiping Liang, Qingjie Zeng, Qing Wei, Xianhua Xie, Chao Yin, Jianzhen Huang

**Affiliations:** College of Animal Science and Technology, Jiangxi Agricultural University, Nanchang, 330045 China

**Keywords:** Fatty liver hemorrhagic syndrome, H3K27me3, ChIP-seq, Transcriptome, Hens

## Abstract

**Background:**

Fatty liver hemorrhagic syndrome (FLHS) in the modern poultry industry is primarily caused by nutrition. Despite encouraging progress on FLHS, the mechanism through which nutrition influences susceptibility to FLHS is still lacking in terms of epigenetics.

**Results:**

In this study, we analyzed the genome-wide patterns of trimethylated lysine residue 27 of histone H3 (H3K27me3) enrichment by chromatin immunoprecipitation-sequencing (ChIP-seq), and examined its association with transcriptomes in healthy and FLHS hens. The study results indicated that H3K27me3 levels were increased in the FLHS hens on a genome-wide scale. Additionally, H3K27me3 was found to occupy the entire gene and the distant intergenic region, which may function as silencer-like regulatory elements. The analysis of transcription factor (TF) motifs in hypermethylated peaks has demonstrated that 23 TFs are involved in the regulation of liver metabolism and development. Transcriptomic analysis indicated that differentially expressed genes (DEGs) were enriched in fatty acid metabolism, amino acid, and carbohydrate metabolism. The hub gene identified from PPI network is fatty acid synthase (*FASN*). Combined ChIP-seq and transcriptome analysis revealed that the increased H3K27me3 and down-regulated genes have significant enrichment in the ECM-receptor interaction, tight junction, cell adhesion molecules, adherens junction, and TGF-beta signaling pathways.

**Conclusions:**

Overall, the trimethylation modification of H3K27 has been shown to have significant regulatory function in FLHS, mediating the expression of crucial genes associated with the ECM-receptor interaction pathway. This highlights the epigenetic mechanisms of H3K27me3 and provides insights into exploring core regulatory targets and nutritional regulation strategies in FLHS.

**Supplementary Information:**

The online version contains supplementary material available at 10.1186/s12864-024-10270-w.

## Background

Fatty Liver syndrome (FLHS) is a prevalent metabolic disorder observed in laying hens during high production periods. Abnormal accumulation of fat, liver enlargement, and yellowing caused the decrease the laying rate of hens that results in huge economic losses of poultry industry [1]. FLHS is mainly caused by nutritional, genetic, environmental, endocrine and toxicological factors and it has been reported that nutritional factors are the main cause of FLHS in laying hens [2]. FLHS causes huge economic losses in the poultry industry due to its high incidence and mortality rate. However, at present, the pathogenesis of FLHS is not completely clear that may be similar to nonalcoholic fatty liver disease. Furthermore, extended consumption of high-energy and low-protein diets will lead to FLHS disease in laying hens, while many studies have used FLHS modeling induced by high-energy and low-protein diet to investigate the underlying mechanism. The pathogenesis of hepatic steatosis is related to epigenetic changes, and differences in cellular epigenetic status may be a predictive factor for individual susceptibility to hepatic steatosis.

The development of hepatic steatosis is associated with epigenetic alterations. Variations in cellular epigenetic status may serve as a predictive factor for an individual’s susceptibility to hepatic steatosis [3]. Epigenetics, defined as a heritable phenomenon that affects gene expression without changing the underlying DNA sequence, mainly including DNA methylation, posttranslational modifications of histone proteins, and chromatin remodeling [4]. One of the most recognized epigenetic regulations is histone modification, which plays a significant role in the diabetes, FLHS, other diseases by regulating the expression of genes [5]. For instance, carbohydrate responsive element binding protein modulated histone modifications in fatty acid synthase are associated with non-alcoholic fatty liver disease [6]. Gao et al. [7] revealed that high acyl-CoA synthetase short-chain family member (ACSS2) expression under hypoxic conditions lead to increased acetylation of H3K9, H3K27 and H3K56. Such H3 acetylation activates fatty acid synthase, leading to improved fatty acid synthesis. In our previous study, we also found that histone modification of H3K27ac in the nonalcoholic fatty liver disease (NAFLD) can regulate the expression of key genes involved in lipid absorption and excretion, as well as the excessive accumulation of fatty acids in the liver [8].

The trimethylation of lysine 27 of histone H3 (H3K27me3) is a common histone modification that regulates chromatin structure and gene expression. It functions as a repressive epigenetic marker, silencing gene transcription [9, 10]. Increasing studies have indicated that H3K27me3 is involved in liver diseases, including NAFLD, liver fibrosis and hepatitis B [11, 12]. For example, H3K27me3 in the proximal promoter region of CFTR in the Hcy treated liver of mice, but not H3K27me1 and H3K27me2 [13]. hyperhomocysteine inhibits cystic fibrosis transmembrane conductance regulator (CFTR) expression through the interaction between H3K27me3 and DNA methylation, thereby activating autophagy and leading to persistent liver injury [14]. In addition, hepatitis B virus X protein promotes ferroptosis in acute liver failure via EZH2/H3K27me3-mediated inhibition of SLC7A11 [15]. Recent research has shown that the H3K27 methyltransferase Ezh2 associates with H3K27me3 on the proximal promoters of *Wnt* genes and directly represses their expression to facilitate adipogenesis [16]. Nevertheless, the regulatory effect of H3K27me3 on FLHS remains poorly elucidated.

In order to investigate the regulating effect of H3K27me3 on FLHS, we used high-energy and low-protein diet to induce FLHS model. Chromatin immunoprecipitation (ChIP)-seq and RNA-seq were performed to comprehensively analyse hepatic lipid metabolism, the regulatory role of H3K27me3, and differential gene expression in FLHS hens. We emphasised the significant role of H3K27me3 in epigenetic regulation mechanisms that contribute to FLHS susceptibility via nutrition. The current study offers insight into the effects of regulating hepatic methylation on hepatic lipid metabolism in laying hens. It provides a foundation for further research into the pathogenesis of FLHS.

## Results

### Histopathological and pathological differences between the hepatic tissue from the healthy and FLHS groups

**Fig. 1:**
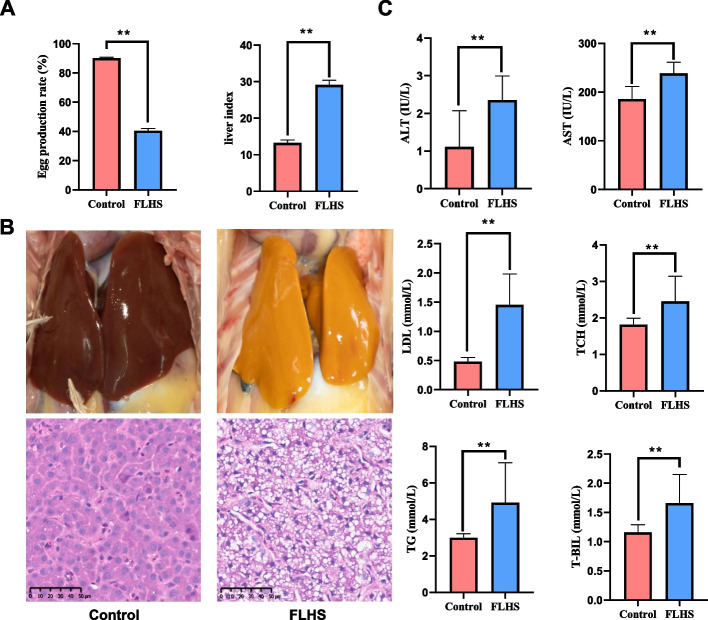
Anatomical and histopathological images of the liver between control group and treatment group (HE, 400×). **A** Egg production and liver index of hens. **B** Hepatic morphological analysis. **C** Determinations of serum biochemical parameters. All data are expressed as mean ± SD (*n* ≥ 25). Values with different capital letter superscripts mean highly significant difference between different groups (*P* ≤ 0.01). Liver index (%) = humid weight of liver/body weight; ALT, alanine aminotransferase; AST, aspartate aminotransferase; LDL, low density lipoprotein; TCH, total cholesterol; TG, triglycerides; T-BIL, total bilirubin

The hens in the FLHS group exhibited symptoms including depression, drowsiness, a pale comb, and a soft and distended abdomen. The egg production was lower than those in the control groups (Fig. 1A). Histological examinations of the liver tissue of the layers in healthy and FLHS groups are shown in Fig. 1. Similar to our previously published results [5, 17], chickens with FLHS had significantly enlarged, thinner and softer livers than healthy chickens. In addition, the colour of the affected livers varied from yellow to orange (Fig. 1B). The healthy control group exhibited normal liver cell architecture without any indications of steatosis or necrosis. In contrast, the group of FLHS subjects exhibited significant fatty degeneration and a substantial accumulation of lipid droplets in their liver cells (Fig. 1B). Significant increases were observed in aspartate aminotransferase (AST), alanine aminotransferase (ALT), low density lipoprotein (LDL), total cholesterol (TCH), triglycerides (TG), and total bilirubin (T-BIL), decreased high density lipoprotein (HDL) in hens of the FLHS group (Fig. 1C). These results suggest that FLHS was successfully established in the present study.

### Genome-wide maps of the histone modification signal of H3K27me3 in healthy and FLHS hens

**Fig. 2:**
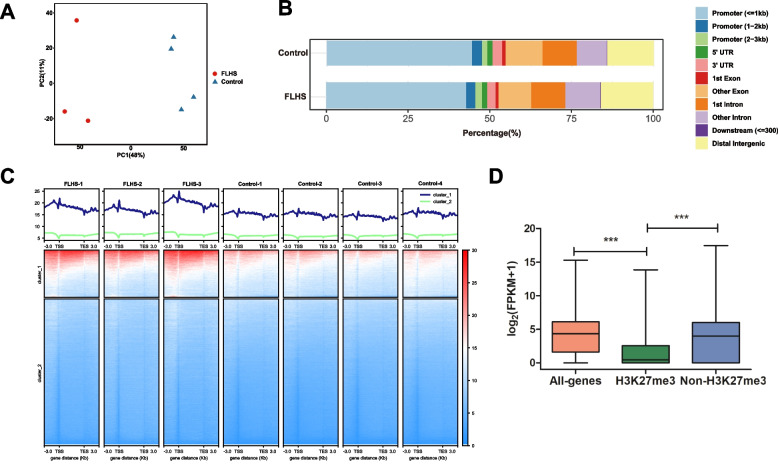
Changes in and expression regulation by H3K27me3 under FLHS. **A** Principal component analysis (PCA) was performed on ChIP-seq data for liver tissues in FLHS (red) and control (blue) groups. First principal component (PC1) accounts for largest possible variance in dataset. Second principal component (PC2) was also calculated, under the condition that it is uncorrelated with (i.e., perpendicular to) PC1. **B** The proportion of genomic features of H3K27me3 peaks was compared with genomic features. Distal intergenic regions refer to genomic regions except downstream (≤300) and promoter regions. **C** Metaplot of normalized H3K27me3 signal over the gene body of protein-coding genes in control and FLHS hens spanning from –3 kb upstream of TSS to + 3 kb downstream of the TES. **D** Gene expression levels as a function of H3K27me3 modification in FLHS hens. *P* values were calculated by a Student’s t test. ***P < 0.001; **P < 0.01; *P < 0.05

In order to comprehensively determine the genome-wide cis-regulatory profiles of healthy and FLHS hens, we conducted ChIP-seq analysis related to the H3K27me3 epigenetic mark. High-quality H3K27me3 ChIP-seq data were generated from postmortem liver tissues of four healthy and three FLHS hens. After alignment, 97.7–98.3% of H3K27me3 reads were mapped unambiguously to the reference chicken genome (GRCg7b), and the replicates showed high experimental reproducibility, as evidenced by the high Pearson correlation coefficient (PCC) values (Table S2 and Fig. S1). Moreover, the livers from the control and FLHS groups can be well segregated by principal component analysis (PCA) of the overall H3K27me3 distributions (Fig. 2A). After data processing, we identified 6214 and 9472 peaks for the control and FLHS hens. The genomic binding profile under control and FLHS conditions was very similar, with approximately 22% of the binding taking place in the intergenic regions (Fig. 2B). We then plotted normalized ChIP-seq read counts across ±3 kb of the transcriptional start sites (TSS) and transcriptional end sites (TES) of all genes. We found alley-like shapes of H3K27me3 around the TSS and TES of genes in control and FLHS hens (Fig. 2C). And there was higher enrichment in the FLHS groups (Fig. 2C). The results showed that more genes in the FLHS hens had H3K27me3 modifications, and that the majority of the regions of modification were in the distal intergenic region and throughout the entire gene body (Fig. 2B and S2). Upon further analysis of gene expression, it was discovered that genes marked with H3K27me3 exhibited significantly lower expression levels than genes without the modification in FLHS hens (Fig. 2D). The results suggest that H3K27me3 may serve as a marker of silencers. Additionally, the H3K27me3-marked elements may interact with promoters of distal target genes, leading to the repression of gene expression and regulation of FLHS processes.

### The analysis of differential H3K27me3 peaks associated with FLHS revealed significant changes in the epigenetic landscape

To validate the functional relevance of the H3K27me3 marked chromatin activation, we next compared the differential histone modification regions between controls and FLHS hens, and also identified 1039 differentially distributed H3K27me3 peaks. We further found that a large proportion of genes (1035 of 1039) were associated with significantly up-regulated H3K27me3 peaks in the hepatic of FLHS-affected hens (Table S3). The KEGG pathway enrichment analysis of these 1035 genes was then performed to identify their physiological functions. In total, 18 pathways were enriched for the genes associated with up-regulated peaks in FLHS hens, such as ECM-receptor interaction (*p* = 0.001), adherens junction (*p* = 0.040), focal adhesion (*p* = 0.011), cell adhesion molecules (*p* = 0.040), MAPK signaling pathway (*p* = 0.003), and TGF-beta signaling pathways (*p* = 0.026) (Table S4). Among the 18 enriched KEGG pathways, the ECM-receptor interaction was significantly enriched. A previous study has shown that ECM remodeling is associated with the progression of hepatic fibrosis [18]. This suggests that genes related to the ECM-receptor interaction may be regulated by differential methylation, further influencing these processes and promoting the development of FLHS. In addition, we aimed to identify sequence motifs for DNA-binding proteins that were significantly enriched for H3K27me3 following FLHS. We detected 23 significantly enriched motifs of hypermethylated peaks modified by H3K27me3. These motifs were predicted to bind to the well-known transcription factors such as LRF, THRB, MYB41, TBX5, and FOXA2, as well as a few uncharacterized motifs (Table 1). These factors have been reported to play roles in regulating the liver development and metabolism.

**Table 1:** Top ten enriched motifs discovery by HOMER for FLHS differential H3K27me3

Motifs	Names	*p*-value	%Targets	%Background
	LRF	1e-3	26.97%	22.54%
	THRB	1e-2	45.38%	40.37%
	MYB41	1e-2	2.79%	1.43%
	TBX5	1e-2	40.75%	36.00%
	FOXA2	1e-2	4.34%	2.93%
	MS188	1e-2	3.47%	1.99%
	MYB67	1e-2	8.29%	5.94%
	SND3	1e-2	4.34%	4.34%
	AT2G15740	1e-2	5.30%	2.82%
	SMB	1e-2	5.88%	4.15%

### Integrating histone methylation and transcriptome changes in FLHS with peak–gene correlation

**Fig. 3:**
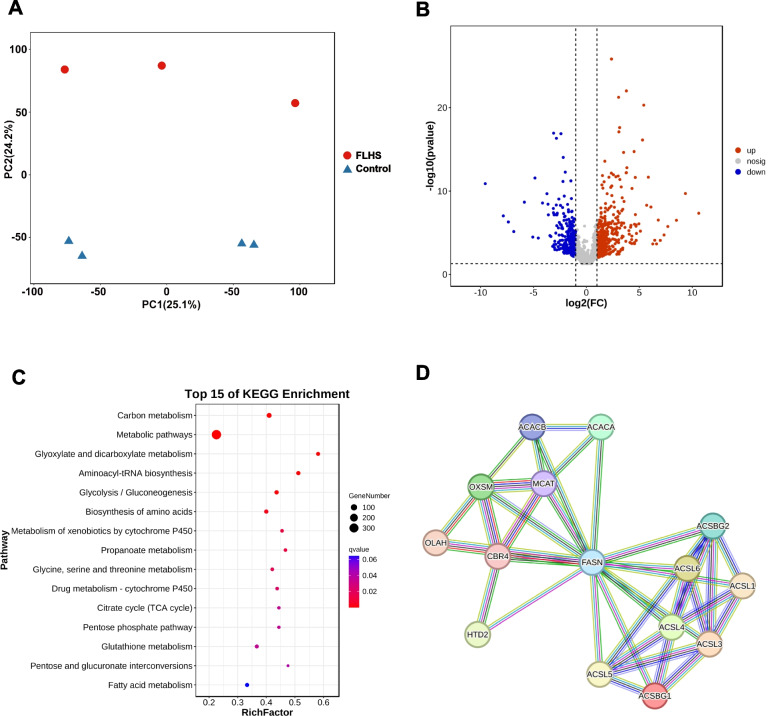
Transcriptome analysis of the livers from FLHS and control hens. **A** PCA of the transcriptomes of the livers from FLHS (red) and control (blue) groups. **B** Volcano plot displaying the transcript levels of differentially expressed genes (DEGs) between the control and FLHS hens. **C** The KEGG pathway enrichment of the DEGs. **D** PPI network of proteins coded by the up-regulated DEGs related to fatty acid biosynthesis in FLHS. The hub gene was in the center of the network. Each node represent proteins coded by the DEGs, and the inter-node connection lines represent the types of protein-protein interactions. The thickness of the solid line represents the strength of the relationship

To investigate systematic differences in the transcriptome landscape between normal and FLHS hens, we performed the hepatic RNA-seq of hens fed with a standard diet and a high-energy and low-protein (HELP) diet, with 5.74 ~ 7.39 Gb RNA-seq data for each sample (Table S5). The sequencing data summary showed that each library had highly consistent statistical parameters (Table S5). PCA was conducted on the gene expression landscape, which resulted in the organization of the replicates into two distinct groups (Fig. 3A). To investigate differentially expressed genes (DEGs) in FLHS hens, we conducted pairwise differential expression analysis between the two groups. A total of 673 DEGs were identified in the two groups, among which 379 genes were up-regulated and 294 genes were down-regulated in the FLHS groups (Fig. 3B and Table S6). The KEGG pathway results demonstrated that DEGs were mainly involved in metabolic pathways, including fatty acid biosynthesis, biosynthesis of amino acids, protein export, and PPAR signaling pathway (Table S7). The majority of above KEGG pathways were categorized as lipid, amino acid, and carbohydrate metabolism, indicating active dynamics of metabolism in FLHS group (Fig. 3C). These results agreed well with previously reported results that abnormally elevated acetylation modifications affect the activity of genes involved in fatty acid metabolism, the TCA cycle, ribosome function, and fatty acid oxidation [19]. This exacerbates hepatic lipid accumulation and metabolic disorders in FLHS. To investigate the interactions between the proteins coded by the DEGs in the FLHS group, a PPI network was constructed using the STRING database and presented through Cytoscape. For the up-regulated DEGs, we found the ECM-receptor interaction with the highest strength values (Table. S8). Consistent with the above upregulation of peak-associated genes enriched to pathway. Interestingly, for the down-regulated DEGs, we found the fatty acid biosynthesis pathway with the highest strength values (Fig. 3D). The hub gene identified from this PPI network is fatty acid synthase (*FASN*), which served as an indispensable enzyme in the de novo synthesis of endogenous long-chain fatty acids [20]. Therefore, the expression of *FASN* in the FLHS group showed an increasing trend. This may be one of the reasons for the higher degree of fatty acid unsaturation in the FLHS group.

**Fig. 4:**
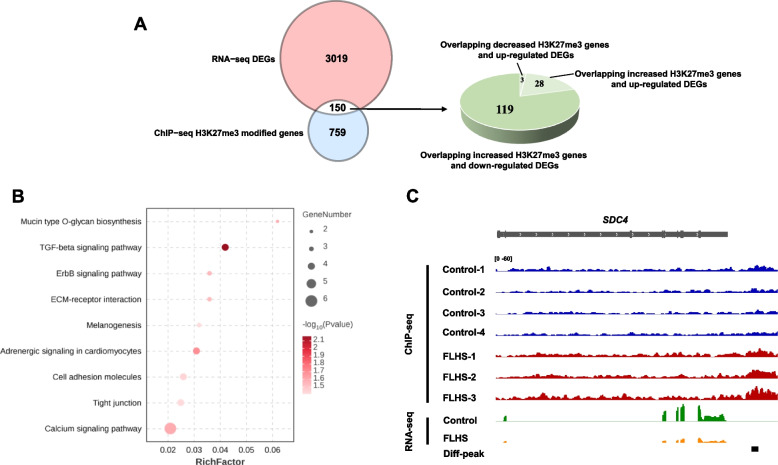
Integration of ChIP-seq and RNA-seq data to identify DEGs with differential H3K27me3 levels. **A** Venn diagram showing the overlap of DEGs from RNA-seq data and differential H3K27me3–associated genes from ChIP-seq data. A set of 150 genes in the overlapping regions were identified including 119 down-regulated DEGs with increased H3K27me3 levels and 31 up-regulated DEGs with decreased H3K27me3 levels. Significantly up- and down-regulated genes are highlighted in different colors according to log2^(fold change)^. **B** Volcano plot showing the transcript levels of DEGs between FLHS and control hens. **C** Functional analysis of genes significantly changing in both up-regulated H3K27me3 modifications and gene expression. The rich factor is the ratio of the number of DEGs annotated in a given pathway to the total number of all genes annotated in the pathway. A higher rich factor indicates greater intensity. The size of the circle indicates the number of genes. **D** Representative genome tracks of RNA-seq and ChIP-seq data for *SDC4* (syndecan 4). Y axis values show normalized read counts

We combined ChIP-seq with RNA-seq to determine the relationship between changes in histone H3K27me3 and gene expression. We analyzed the intersection of the DEGs and the H3K27me3 histone modifications and observed that 150 DEGs may be potentially regulated by H3K27me3 in hens. Among these, 79.3% (119 of 150) genes were down-regulated and 20.7% (31 of 150) genes were up-regulated in FLHS groups. Interesting, all down-regulated genes gained H3K27me3, whereas only 9.7% (3 of 31) of up-regulated DEGs displayed a loss of H3K27me3 marker (Fig. 4A). The remaining 90.3% (28 of 31) showed up-regulated DEGs with increased H3K27me3. This result suggests a strong negative correlation between H3K27me3 modification and gene expression during the development of FLHS. Further evaluation of these 119 upregulated DEGs with H3K27me3 loss revealed the significant enrichment in ECM-receptor interaction, cell adhesion molecules, tight junction, adherens junction, and TGF-beta signaling pathways (Fig. 4B). Specifically, many genes, such as syndecan 4 (*SDC4*), integrin beta 4 (*ITGB4*), and junctional adhesion molecule 3 (*JAM3*), showed decreased expression and enrichment of H3K27me3 marks around each locus in FLHS compared to control hens (Fig. 4C). These are all key mediators of the cell adhesion, migration, proliferation, endocytosis and mechanotransduction [21]. Hepatocytes are equipped basically with a vast variety of the junctions, including anchoring junctions, tight junctions, adherens junctions, and gap junctions to maintain anatomical organization [22–24]. Integration of the ChIP-seq data with the transcriptome indicated that the H3K27me3 modification may have a systemic impact on the expression of genes that are associated with maintaining tissue integrity and cellular interactions during the development of FLHS.

## Discussion

The liver is the primary organ responsible for lipid metabolism in poultry. It plays a crucial role in various processes related to lipid metabolism, such as fatty acid absorption, de novo synthesis, and oxidative decomposition of fatty acids. The disease may be similar to that of NAFLD in humans [25]. The theory is that Insulin Resistance (IR) induces the accumulation of large amounts of free fatty acids (FFA) in the liver. Additionally, oxidative stress causes an increase in reactive oxygen species in the liver, and lipid peroxidation produces lipid peroxides. These lipid peroxides can increase endogenous peroxides and inhibit antioxidant activity, both of which further aggravate liver cell damage [26]. Abnormal expression of genes involved in the above biological processes will lead to FLHS.

Covalent chromatin modification refers to modifications on non-coding DNA regions that interact with histone molecules, such as methylation and acetylation. These modifications can impact the structure and stability of chromatin, ultimately influencing gene expression [27]. The histone modification H3K27me3 is an epigenetic mark that represses gene expression, however, the mechanisms through which histone modifications regulate hepatic lipid changes from normal to FLHS stages in laying hens remain unclear. To address this issue, we constructed hens affected by FLHS through the simulation of a diet that is high-energy and low-protein, which is commonly used in production practices. Subsequently, we present the changes in H3K27me3 profiles in the liver tissues of FLHS-induced hens compared to the control groups. First, we noticed the distribution of H3K27me3 was enriched around the distal intergenic region and the gene body (Fig. 2B), which is consistent with the patterns observed in insects and mammals [28, 29]. Second, the FLHS group exhibits a significantly higher enrichment of ChIP-seq reads for H3K27me3 compared to the control (Fig. 2C), suggesting that more genes in the FLHS hens had H3K27me3 modifications. Third, H3K27me3 is typically associated with the repression of gene expression [30], and a significant negative association was found between the presence of H3K27me3 and expression levels in FLHS hens (Fig. 2D). The results suggest that H3K27me3 may serve as a marker of silencers. Additionally, the H3K27me3-marked elements may interact with promoters of distal target genes, leading to the repression of gene expression and regulation of FLHS processes.

We think that some genes can have both activating trimethylated Histone H3 Lysine 4 (H3K4me3) and repressive H3K27me3 histone modifications simultaneously. These regions are called bivalent domains and are thought to maintain genes in a poised state, ready for activation or repression depending on cellular cues. Perhaps this is why so many genes are associated with significantly up-regulated H3K27me3 peaks in the liver of FLHS-affected hens. We will follow up with further studies on the H3K4me3 modification of FLHS.

Transcription factors (TFs) act as direct activators of transcription and may also play a role in inducing epigenetic modifications in specific regions of the genome [31]. This study revealed TFs associated with hypermethylated peaks of H3K27me3, such as LRF, THRB, MYB41, TBX5, and FOXA2 (Table 1), which are known to regulate liver development and metabolism. In obese and high-fat diet states, insulin-mediated FOXA2 repression has been shown to be the molecular mechanism linking lipid-based abnormalities to metabolic disorders. This leads to elevated triglycerides and reduced plasma high-density lipoprotein (HDL) levels [32]. TBX5 encodes a transcription factor that plays a crucial role in the differentiation of multiple organs during embryogenesis. Genetic variants in TBX5 may lead to impaired development of liver tissue [33]. Chaves et al. demonstrated that the nuclear receptor THRB plays a significant role in hepatic steatosis, highlighting the influence of thyroid hormone on lipid metabolism in the liver [34]. The results indicate that DNA-binding proteins enriched by FLHS-related differential H3K27me3 peaks are primarily involved in regulating liver metabolic balance. This is consistent with the fact that transcription factors can act as activators of transcription and play a critical role in regulating transcriptional programs that control disease phenotypes [35].

To study the molecular mechanisms underlying the differences in liver response to FLHS, we generated comparative transcriptomic analyses between the healthy and FLHS hens. We found that DEGs are mainly enriched in fatty acid, amino acid and glucose metabolism (Fig. 3C), which are essential for the maintenance of egg-laying activity in hens. Of primary interest is the fatty acid metabolism in hens with FLHS. The study showed that genes related to fatty acid synthesis and lipid transport, such as fatty acid synthase (*FASN*), acetyl-CoA carboxylase (*ACAC*), and long-chain acyl-CoA synthetase (*ACSL*), actively participated in these metabolisms. *FASN* serves as a central regulator of lipid metabolism and is the primary enzyme responsible for the anabolic conversion of dietary carbohydrates into fatty acids [36]. ACC is an enzyme that catalyzes which catalyze the carboxylation of acetyl-CoA to produce malonyl-CoA, which in turn is utilized by FASN to produce long-chain saturated fatty acids [37]. ACSL activates both the synthesis of cellular lipids and long-chain fatty acids [38]. Several studies have shown that the desaturation and elongation of fatty acids contribute to the pathogenesis of NAFLD [39, 40]. This suggests that FLHS affects the catalytic activity of enzymes involved in fatty acid metabolism, resulting in the elevation of long-chain saturated fatty acids. In addition to lipid metabolism, disorders of glucose metabolism contribute to the pathogenesis of NAFLD [41]. In the present study, we also found DEGs enriched in all types of glucose metabolism, such as glycolysis/gluconeogenesis, pyruvate metabolism, pentose phosphate pathway, galactose metabolism, citrate cycle and so on. Malate dehydrogenase (*MDH1*), pyruvate dehydrogenase (*PDHA*), and aconitate hydratase (*ACO*) are involved in the conversion of NADP^+^ to NADPH through the TCA cycle [42]. Oxidative phosphorylation is the primary source of energy for the cell’s aerobic activities and the main pathway for ATP production [43]. The results indicate that the disturbance of electron respiratory chain complex activity affects the conversion of NADPH to ATP, which provides new insight into the pathogenesis of FLHS. Also, amino acid metabolism is closely associated with hepatic lipidosis. In this study, the disorder of amino acid metabolism was observed in FLHS layers. Therefore, metabolic pathway disturbances may be the cause of the inflammation that occurred in FLHS. The alterations in liver metabolites and metabolic pathways may provide evidence to understand the pathogenesis of FLHS. Moreover, we observed that DEGs were also enriched in peroxisome proliferator-activated receptor (PPAR) signaling pathway. In the previous study, it was found that the PPAR signaling pathway was disrupted, leading to fat accumulation in the liver of laying hens with FLHS. The PPAR signaling pathway is widely considered a key target for lipid metabolism, and inhibiting it could reduce hepatic fat accumulation, thereby relieving fatty liver [44]. Therefore, the disruption of the PPAR pathway may play a critical role in the development and advancement of FLHS.

**Fig. 5:**
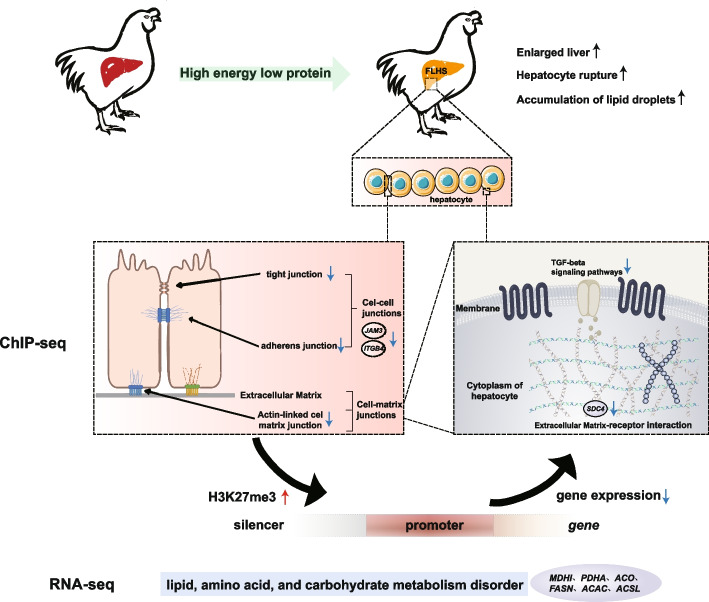
A proposed model on the epigenetic regulation mechanisms of histone H3K27me3 causing the FLHS in laying hens. The modification of H3K27me3 histone on genes involved in maintaining of tissue integrity and cellular interactions may regulate lipid anabolism, resulting in increased lipid accumulation in the liver tissue of hens, and ultimately leading to the development of FLHS

It is widely recognized that reprogramming of epigenetic states is essential for establishing and maintaining lineage-specific transcriptional programs. Therefore, to fully comprehend the role of epigenetics in FLHS, it is necessary to conduct an integrative analysis of both the transcriptome and the methylome. We found a positive correlation between H3K27me3 density and down-regulated gene expression, and genes annotated with H3K27me3 peaks were more likely to be repressed (Figs. 2D and 4A). Interestingly, we found hypermethylated and down-regulated genes were involved in ECM-receptor interaction, tight junction, cell adhesion molecules, adherens junction, and TGF-beta signaling pathways (Fig. 4B), suggesting a potential impact on hepatic integrity. The TGF-β signaling pathway is known to make most hepatic cell types susceptible [45]. On the other hand, a recent study by Waddell et al. [46] found that the TGFβ-ECM-Integrin signaling axis adjusts the expression of a fibronectin-rich extracellular matrix to promote polycystic liver disease. The ECM comprises various families of molecules, such as collagens, non-collagenous glycoproteins, glycosaminoglycans, and proteoglycans [47]. It plays a crucial role in the processes involved in the development and maintenance of organisms. Several studies have shown that ECM-receptor interaction plays a critical role in liver fatty acid metabolism [48, 49], affecting it by regulating key TFs [50]. Zhang et al. (2023b) found that the liver may regulate lipid metabolism in abdominal and subcutaneous adipose tissue through ECM-receptor interactions and metabolic pathways, specifically fatty acid metabolism and unsaturated fatty acid synthesis. These interactions also provide physical support that facilitates adipocyte proliferation, differentiation, and migration [51–53]. Furthermore, studies on chickens with varying dietary intake have reported that the ECM-receptor interaction pathway plays a significant role in lipid metabolism in the liver and abdominal adipose tissue [54, 55]. In addition, Hernandez-Guerra et al. demonstrated that impaired intercellular communication and gap junction were involved in the fatty liver pathological process, with gap junction playing a protective role by maintenance of homeostasis through cell-to-cell communication [56]. Gap junctions play a protective role by maintaining homeostasis through cell-to-cell communication. The deregulation of the expression and function of tight junctions dismantles the architecture of the hepatic parenchyma, leading to liver diseases [57]. In addition, hepatocyte rupture, enlarged liver and a haemorrhagic phenotype were observed in FLHS hens (Fig. 1). Therefore, we assumed that the dysfunction of cellular junctions and communication pathways, as well as vascular contraction, may be attributed to the disrupted structure of hepatocytes and blood vessels. In the current study, we proposed a model involving H3K27me3 in epigenetic regulation of FLHS in laying hens (Fig. 5). Taken together, our results indicate that the modification of the H3K27me3 histone on genes involved in maintaining of tissue integrity and cellular interactions may regulate lipid anabolism, resulting in increased lipid accumulation in the liver tissue of hens, and ultimately leading to the development of FLHS.

## Conclusions

Overall, H3K27me3 has been shown to have significant regulatory function in FLHS, mediating the expression of crucial genes associated with the ECM-receptor interaction pathway. This highlights the epigenetic mechanisms of H3K27me3 and provides insights into exploring core regulatory targets and nutritional regulation strategies in FLHS.

## Materials and methods

### Preparation of animals and samples

A total of 90 Hy-Line Brown layers (an average body weight of 1.5 kg, age: 150 days) were purchased from local commercial farm used in the experiment. The hens were housed at conditions as follows: temperature (28–32 °C), humidity (50–75%), light time 16 L:8D (Lighting was controlled daily between 6 a.m. and 10 p.m.), and free access to food and drinking water. After 1 week of acclimation, the hens were randomly assigned into two treatments including Control fed with basal diet (Con) and the disease group fed with a high-energy, low-protein diet (FLHS). Layers in the control group were fed a standard diet and those of the experimental group were fed a HELP diet (Table S1). The hens were raised in ladder cages with each 45 hens in each a treatment and 2 hens in each cage. After 80 days of feeding, the hens were weighed and their egg production rate was evaluated. They were sacrificed to collect the samples. The hens were fasted for 12 h. Then, 25 hens were randomly selected from each subgroup to collect blood specimens from the left-wing vein. After that, they were euthanized by carbon inhalation. The liver, abdominal lipid and stomach lipids were weighed. The liver samples (right lobe of liver) were collected and stored in 4% neutral polyformaldehyde fixative for histological analysis or frozen in liquid nitrogen for ChIP-seq or RNA-seq analysis. The others were collected frozen in − 20 °C refrigerator to subsequent analyze.

### Determination of serum biochemical indexes in hens

According to the manufacturer’s protocol, the level of Triglyceride (TG), Total Cholesterol (TC), High Density Lipoprotein (HDL), Low Density Lipoprotein (LDL), Alanine Aminotransferase (ALT), Aspartate Aminotransferase (AST), Total Bilirubin (T-BIL), Albumin (ALB), Direct Bilirubin (D-BIL) and Total Protein (TP) in serum were determined by a colorimetric method using an automatic biochemical analyzer (Hitachi, Ltd., Beijing, China) using relevant kits (Medicalsystem Biotechnology Co., Ltd., Ningbo, China).

### Histological analysis

The Liver tissues were fixed in 4% paraformaldehyde, then embedded in wax and sectioned at 4 μm for haematoxylin and eosin (H&E) staining. The stained sections were observed under a light microscope and the image was taken using white light scanner (Hamamatsu Photonics KK Co., Ltd., Tokyo, Japan).

### RNA sequencing (RNA-seq) and data analysis

Total RNA of the above liver samples was extracted using TRIzol (Takara, Dalian, China) by following the manufacturer’s protocol. Then, they were delivered to Novogene Bioinformatics Technology Co., Ltd. (Beijing, China) for a commercial service. RNA integrity was assessed using the RNA Nano 6000 Assay Kit of the Bioanalyzer 2100 system (Agilent Technologies, CA, USA). According to the manufacturer’s instructions, the clustered samples were sequenced on an Illumina Novaseq platform and 150 bp paired-end reads were generated in the library.

Sequenced raw data were qualified, filtered, built, and mapped to the chicken (*Gallus gallus*) genome GRCg7b (Ensembl release 109) using Hisat2 v2.0.5 [58]. Principal component analysis (PCA) was performed using an online software (https://www.omicshare.com/ tools/). Differentially expressed genes (DEGs) were identified using DESeq2 R package (1.20.0) [59], with *P-value* < 0.05 and |Log_2_^fold change^| > 1. The independent sequenced samples were mapped to the reference genome and determined expression profiles as fragments per kilobase of transcript per million mapped reads (FPKM) by RSEM v1.3.040 [60]. For Kyoto Encyclopedia of Genes and Genomes (KEGG) pathway enrichment analysis, we first compiled a KEGG term list for all protein-coding genes in the *G. gallus* genome. We used eggNOG 5.0 [61] to search for orthologous genes in the eggNOG database. The DEGs were then used as input to the universal enrichment protocol, which can be accessed through the online platform (https://www.omicshare.com/tools/).

### Chromatin immunoprecipitation (ChIP)-sequencing analysis

Chromatin samples were performed according to SimpleChIP® Plus Enzymatic Chromatin IP Kit#9005 kit (Cell Signaling Technology, Boston, USA) instructions. Briefely, the liver samples were treated with 14% formaldehyde to cross-link proteins covalently to DNA. After the cross-linked cells were destroyed and the chromatin was cut into smaller fragments using ultrasound. Anti-H3K27me3 (Cell Signaling Technology, Boston, USA) was added to the supernatant and the sequencing libraries were then sequenced using an Illumina NovaSeq 2500 platform by a commercial service provided by ANOROAD (Beijing, China). Fraction of reads in peaks (FRiP) which evaluates the number and strength of peaks obtained for each ChIP replicate were used to measure the signal-to-noise ratio (S/N). We considered an immunoprecipitation reaction successful when its FRiP was at least 0.01.

The sequenced reads were then trimmed and aligned to the chicken (*Gallus gallus*) genome GRCg7b (Ensembl release 109) using Bowtie2 (v 2.5.1) [62]. Samtools (v 1.3.1) was used to remove potential PCR duplicates [63]. To perform peak calling for each replicate, we used the ‘ChIP-seq’ function, implemented in MACS2 v2.1.1 [64], with input group as the control. We used the ‘callpeak’ module with the following parameters: --nomodel --broad --bdg. We then identified the overlapping peaks between both replicates using the ‘findOverlapsOfPeaks’ function in the R package ChIPpeakAnno v3.10.2 [65]. Diffbind (v 2.14.0) [66] was used to identify differentially peaks. The downstream analysis was performed on an overlap peak in at least two samples, and these were merged to form a consensus peak set. Then, the differential peaks were calculated by DESeq2. The screening thresholds were designed as *P*-value < 0.05 and |Fold change | > 1.5. The distance from the peak to the TSS of the nearest gene is calculated by ‘annotatePeak’ function in ChIPseeker [67]. A 3.2 kb (up: 3000 bp down: 200 bp) sequence near the TSS was selected as a promoter region. All alignment results were then converted to coverage bigwig files and normalized to the corresponding input using DeepTools (v 2.5.0) [68]. The bigwig formats can be visualized by software IGV (Integrative Genomics Viewer) [69]. The module “computeMatrix” in DeepTools was used to generate reads abundance from all ChIP-seq datasets around − 2 kbp to TSS, TSS to TES and TES to + 2 kb. These matrices were then used to create heatmaps, using the deepTools commands “plotHeatmap”. Subsequently, Hypergeometric Optimization of Motif Enrichment (HOMER) (v 3.1) was conducted for identifying TF binding motifs enriched by differential peaks with the command of findMotifsGenome.pl [70].

### Protein–protein interaction network

The database of Search Tool for Retrieval of Interacting Genes (STRING) is an online database for predicting PPIs [71]. By utilizing STRING (v 10.0, http://www.string-db.org/) database, the PPIs of genes were analyzed. The protein pairs with PPI score > 0.4 were collected. Then, Cytoscape (v 3.2.0, http://www.cytoscape.org/) was used to visualize the predicted PPI network [72].

### Supplementary Information

**Supplementary Material 1.**

**Supplementary Material 2.**

**Supplementary Material 3.**

**Supplementary Material 4.**

**Supplementary Material 5.**

**Supplementary Material 6.**

**Supplementary Material 7.**

**Supplementary Material 8.**

**Supplementary Material 9.**

**Supplementary Material 10.**

## Data Availability

The authors declare that the data supporting the findings of this study are available within the article and its supplementary information files. All the raw sequences have been deposited in the NCBI database Sequence Read Archive with the BioProject number PRJNA1081611.
