# Supplementary material for: Epigenetic regulation of H3K27me3 in laying hens with fatty liver hemorrhagic syndrome induced by high-energy and low-protein diets
Source: BMC Genomics. 2024 Apr 16;25:374. doi: 10.1186/s12864-024-10270-w (PMC11022457; doi:10.1186/s12864-024-10270-w)
Supplement: Supplementary file 1 — Supplementary Material 1. [file 12864_2024_10270_MOESM1_ESM.docx]

**Table S1. The diet composition formula and nutritional level**

| HELP diet | | Control group (%) | FLHS group (%) |
| --- | --- | --- | --- |
| Ingredient | Corn | 64.50 | 72.50 |
|  | Soybean meal | 23.50 | 10.90 |
|  | fat-Soybean oil | - | 5.20 |
|  | Fish meal | 1.50 | 1.02 |
|  | CaHCO_3_ | 1.50 | 1.50 |
|  | Limestone | 8.00 | 8.00 |
|  | Salt | 0.30 | 0.30 |
|  | Lys | 0.10 | 0.05 |
|  | Met | 0.20 | 0.14 |
|  | 50% Cholin (mg/kg) | 0.10 | 0.09 |
|  | Premix* | 0.30 | 0.30 |
|  | Total | 100 | 100 |
| Nutrient level |  |  |  |
|  | CP | 16.50 | 11.10 |
|  | Lys | 0.93 | 0.55 |
|  | Met | 0.48 | 0.35 |
|  | Thr | 0.6 | 0.40 |
|  | Ca | 3.52 | 3.46 |
|  | TP | 0.61 | 0.54 |
|  | AP | 0.36 | 0.33 |
|  | （MJ/kg） | 11.56 | 13.10 |
|  | Caloric Protein Ratio | 70 | 118 |

* Premix was composed of the following per kg diet：pyridine chromium, 2.00 mg; Mn, 25.00 mg; KI, 4.00 mg; antioxidant, 2.00 mg; Cu, 2.50 mg; choline, 500.00 mg; sodium selenite, 0.60 mg; CoCl, 1.20 mg; kininase, 7.50 mg; Zn, 85.00 mg; vitamins, 150.0 mg; Fe, 100.0 mg; phytase, 10.00 mg; betaine, 150.0 mg; Ca_3_PO_4_, 7.00g
