# Supplementary material for: Epigenetic regulation of H3K27me3 in laying hens with fatty liver hemorrhagic syndrome induced by high-energy and low-protein diets
Source: BMC Genomics. 2024 Apr 16;25:374. doi: 10.1186/s12864-024-10270-w (PMC11022457; doi:10.1186/s12864-024-10270-w)
Supplement: Supplementary file 9 — Supplementary Material 9. [file 12864_2024_10270_MOESM9_ESM.pdf]

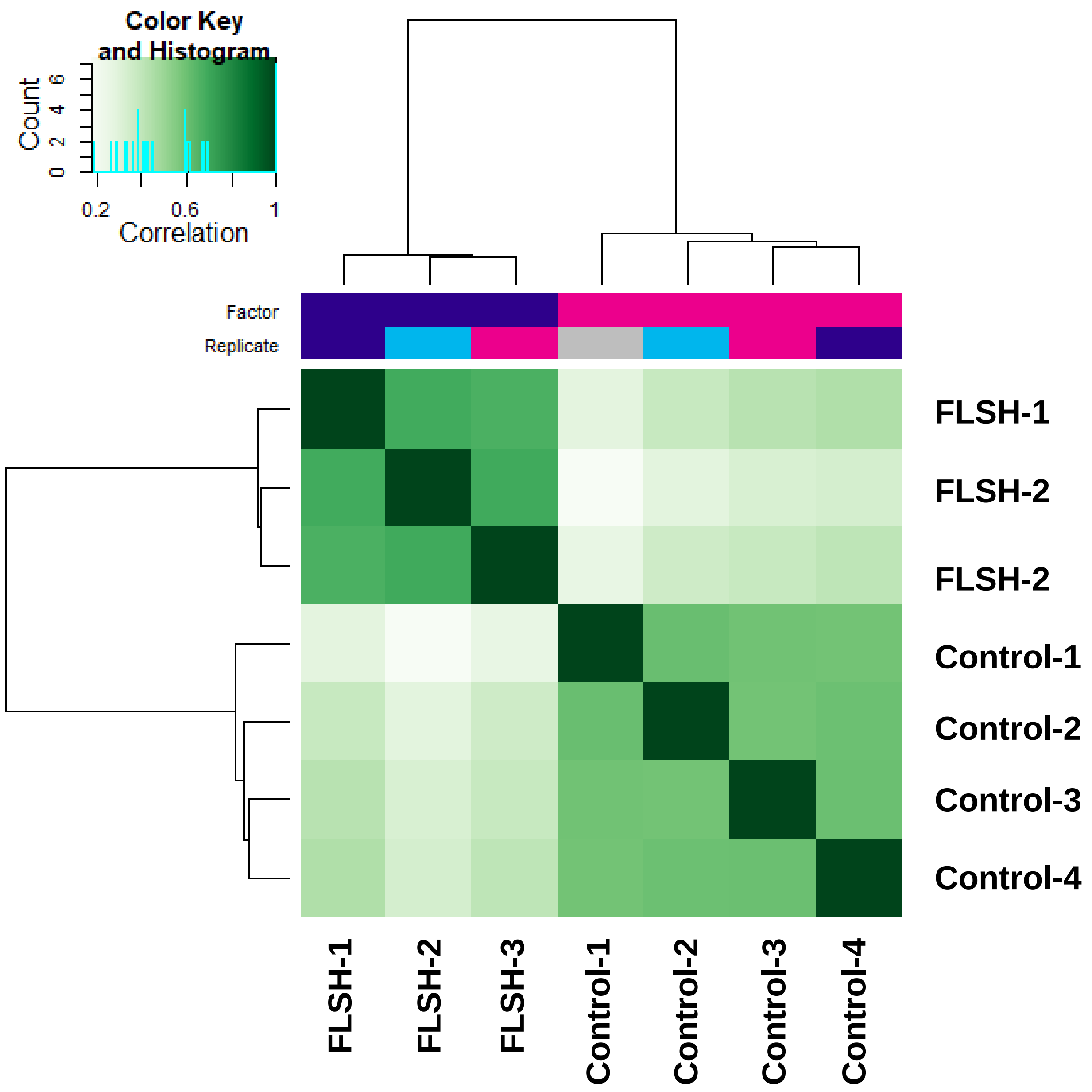

Supplementary Figure 1. Pearson correlation coefficient (PCC) of the mapped read counts in H3K27me3 replicates.
