# Supplementary material for: Epigenetic regulation of H3K27me3 in laying hens with fatty liver hemorrhagic syndrome induced by high-energy and low-protein diets
Source: BMC Genomics. 2024 Apr 16;25:374. doi: 10.1186/s12864-024-10270-w (PMC11022457; doi:10.1186/s12864-024-10270-w)
Supplement: Supplementary file 10 — Supplementary Material 10. [file 12864_2024_10270_MOESM10_ESM.pdf]

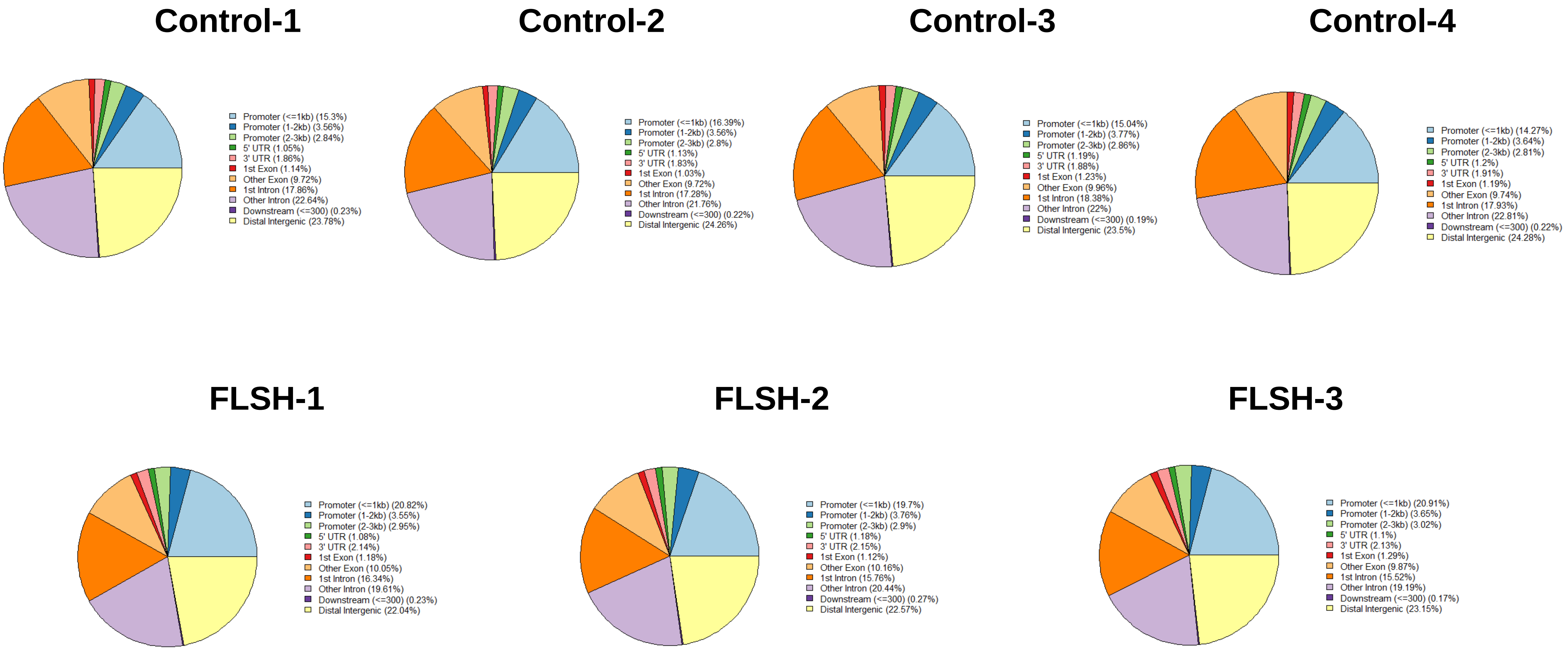

Supplementary Figure 2. The proportion of genomic features of H3K27me3 peaks compared with genomic features.
